# Family health partners in regional network structures (NEST): A non-randomized controlled trial among parents of chronically ill and disabled children

**DOI:** 10.1371/journal.pone.0288435

**Published:** 2023-07-17

**Authors:** Stefan Nickel, Isabella Helmreich, Jan Broll, Daniel Lüdecke

**Affiliations:** 1 Institute of Medical Sociology, University Medical Center Hamburg-Eppendorf, Hamburg, Germany; 2 Leibniz Institute for Resilience Research, Mainz, Germany; Public Library of Science, UNITED KINGDOM

## Abstract

**Background:**

The overarching project goal is to evaluate the effectiveness of a cross-sectoral and cross-service provider offering professional support for families with chronically ill and disabled children: so-called Family Health Partners (in German: ‘Familien-Gesundheits-Partner’ or FGP). This needs-oriented service, which is anchored in regional networks, aims to provide ‘holistic’ support for families with children in need of care.

**Methods:**

We are carrying out a non-randomized controlled trial with four points of measurement (t0-t3 in 18 months), beginning in January 2022. Both intervention and control group include 102 families. Primary outcome measure is the quality of life, secondary outcomes are resilience factors and associated measures as well as the access to care. Multilevel regression models will be used to analyze the longitudinal data.

**Discussion:**

The strength of this study is that it looks at the health and resilience of all family members involved by examining how a FGP can influence the entire family system with regard to increasing quality of life, resilience and self-efficacy. The network structures of FGP also open up better to previously unknown regional supply offers. There are, however, a number of limitations (e.g. type of outcomes, sample size).

**Trial registration:**

This study was first registered on the German Clinical Trials Register before enrolment of participants started (ID: DRKS00027465, 4 January 2022). In order to promote its dissemination, it was also retrospectively registered on ClinicalTrials.gov (ID: NCT05418205, 14 June 2022).

## Introduction

The NEST project (German acronym for, Strengthening and relief for families with children in need of care through family health partners in regional NEtwork STructures‘) starts facing the problem that families with children with severe health problems due to disability or chronic illness are exposed to strong emotional, social, economic and temporal burdens [1–7]. These burdens result in further health, social and economic risks [8–14], which often lead to fragile living and burdening care situations for the families concerned [15]. This is, among other things, also due to legal regulations that do not do justice to the respective individual life situation and the lack of competent counseling services [16].

The overarching objective of the project is the implementation and evaluation of an innovative support service that addresses the problem. The support by so-called Family Health Partners (in German: ‘Familien-Gesundheits-Partner’ or FGP) aims to provide needs-based, individual counseling for all members in affected families to reach the best possible physical, psychological and participation-oriented care for children in need of care; and thus relieving the family members who spend much time for care and assistance of their child. The intervention to be evaluated here with regard to its effectiveness (processes and structures) was summarized in line with the terms of FGP. This personalized approach, which is embedded in the regional network, aims to provide “holistic” support for families with children in need of care. This means that the needs of the families are defined individually and independent of sectoral or service provider specific offers (structured assessment).

The needs of families not only relate to medical, nursing or therapeutic care, but also to assistance regarding social law, economic and bureaucratic advice as well as addressing social, psychosocial and emotional needs and social participation. Based on the identified need for support, affected families are individually accompanied by FGP over a certain period of time (in the project context: more than four times a year). The medium-term goal of FGP support is to strengthen or preserve the family as a self-help system, i.e. self-efficient, independently acting primary resource for the care and support of their children (preventive).

### Hypotheses

The development and systematic establishment of the three FGP core processes of a) structured assessment of the needs and requirements of families, b) counseling and coordination function and c) opening up accesses to needs-based support and relief offers in the regional supply network greatly contrast with the regular supply:

- better recognition of the needs and requirements of families with children in need of care, across service providers and payers, and thus being addressed in a targeted and timely manner;
- more relief for families with children in need of care, and family care system strengthened;
- improved health, care and quality of life for families and children in need of care.

In the quantitative research part of the project, a prospective design is used to examine whether and to what extent the FGP intervention, coordination and family support as well as network support is suitable for achieving the care goals. Hypotheses are that through the intervention:

- Access to care for families with children in need of care is improved.
- Over-, under- and misuse of health services is avoided.
- The family is strengthened as the most important resource for quality of life.

## Materials and methods

### Design

A non-randomized controlled study (quantitative online survey) over 18 months is ongoing with four measurement points: baseline at the start of the intervention (t0), interim assessments after 6 (t1) and 12 (t2) months, and a final survey after 18 months (t3). In order to be able to identify at least medium effects (Cohen’s d = 0.5) [17] with a test power of 0.80 and an alpha error of 0.05, at least 52 families both from the intervention and control group are required to participate in the study. Since there are hardly any longitudinal intervention studies with particular regional differences in the field of self-help research, the extent of the regional variance (hierarchical data structure) can only be estimated. Assuming 5% intracluster variance explained by the hierarchical data structure, this sample size must be increased by the variance inflation factor [18], resulting in a minimum of 73 families per group. With an estimated drop-out rate of 20–40% based on current studies (e.g. Recapture Life-AYA study [19]), the number of samples ultimately increases to a total of N = 204 families.

The study is conducted with parents and other guardians (all genders and ethnicities) with at least one disabled and/or chronically ill child under 18 years (benefit receipt according to § 37 of Germany’s Social Security Code V and/or level of care > 1), and is limited to the main adult caregivers of the child. The families from the intervention group are recruited via the care service ‘nestwärme gGmbH’ and its network partners in three metropolitan areas of Germany (Trier, Saarbrucken and Munich). The families for the control group are recruited via the umbrella organization ‘Kindernetzwerk e.V.’, which also has regional associations in these regions. In contrast to the control group, which is supported by standard care, the intervention group receives an FGP that carries out a structured assessment in the family and accompanies the family throughout the intervention period. Recruitment for the baseline data collection already started in the intervention areas in January 2022. Control group participants were recruited throughout Germany, with 102 families consenting to take part in the study until the end of September 2022.

### Measurements

For the description of the sample, information on parent’s age, gender, marital status, child’s primary caregiver, education, employment, household income, and place of residence as well as child’s age, gender, diagnosis, level of care, and number of siblings are used.

Primary outcome measurements relate to *family quality of life*. For this purpose, validated questionnaires are used, which include the following dimensions:

- Parents’ mental and physical health (GHQ-28) [20]
- Well-being (WHO-5) [21]
- Stress experience (PSS-4) [22]
- Family burden due to illness of the child (FaBel-20) [23]
- Family cohesion (RSA) [24]
- Resilience (BRS) [25]
- Individual stressor exposure (MIMIS, LEC) [26, 27]

Secondary outcomes of the quantitative survey are:

- *Resilience factors and associated measures*
  - Optimism / positive thinking (SOP-2) [28]
  - Locus of control (IE-4) [29]
  - Self-efficacy (ASKU-3) [30]
  - Social support (OSSS-3) [31]
- *access to care*
  - Satisfaction with health care [15]
  - Satisfaction with health care coordination (from t1) [32]
  - Knowledge and use of support services [33]

To assess possible over-, under- and misuse of health services the results of the process evaluation will be used. For this purpose, all FGPs involved are questioned by means of guideline-based qualitative interviews.

### Statistical analysis

SPSS™ 27 and the programming language R are used as software for the statistical analyzes. In the first step at baseline, the data analysis is primarily descriptive according to sample characteristics and outcome measures. Subgroup analyzes between intervention and control group are made using Chi^2^ test, t-test and Mann-Whitney U test, based on the measurement/scales of the related variables. Multilevel regression models will be used to analyze the longitudinal data. This makes it possible to statistically model and control regional differences. Interaction effects are calculated to analyze group differences (intervention vs. control).

If the proportion of missing values (e.g. "don’t know", no answer) is lower than 5%, these will be excluded from the analysis in accordance with the GESIS guidelines [34]. Else, for larger proportions missing data will be imputed using the multivariate imputation by chained equations method [35].

### Ethics, registration and dissemination

This study was approved by the Local Psychological Ethics Committee at the University Medical Center Hamburg-Eppendorf (authorization number: LPEK-0370). The study was prospectively registered on the German Clinical Trials Register before the enrolment of participants started (ID: DRKS00027465, 4 January 2022), and retrospectively on ClinicalTrials.gov (ID: NCT05418205, 14 June 2022). The authors confirm that all ongoing and related trials for this intervention are registered.

At the beginning of the study, all attendees gave written informed consent on the study and agreed to participate in the baseline and follow-up surveys after 6, 12 and 18 months. The privacy of the participants is guaranteed by storing encrypted data. Every participant will receive a pseudo-anonymous study number. The key is only accessible to the study team and co-workers. Data and material will only be used in coded form within possible collaboration projects.

The results of this study will be made available through peer-reviewed scientific journals and presentations at relevant conferences. Furthermore, a project website was launched to provide information about the current status of the project (https://forschungsprojekt-nest.de/). A handbook with recommendations for the cross-project implementation of sustainable processes and structures for the needs-based care (with special regional consideration, cost and service provider-specific implementation barriers and legal regulation needs) will be developed.

## Discussion

The presentation and analysis of general and/or specific mechanisms of action of a regionally networked organization that operates across sectors, costs and service providers with regard to their effectiveness for families with children in need of care is new. As far as can be seen, only the case management in the German PariSozial project [36] has been subject to a scientific evaluation–albeit under the premise of its effectiveness with regard to ensuring the need for care. The question of the effectiveness of the support networks in relation to multidimensional outcomes has not yet been consistently posed nor answered. Here, multidimensional outcomes include access to care, family health and quality of life, preservation and mobilization of self-help resources and service provider-financed care.

The strength of this study is that it, besides the usual variables of ‘prescribed’ care, looks at the health and resilience of all family members involved by examining how a FGP can influence the entire family system with regard to increasing quality of life, resilience and self-efficacy. The network structures of FGP also open up better to previously unknown regional supply offers (incl. youth welfare office, school, social pediatric centers etc.). In addition, the comprehensive information collected in this study will also enable us to answer other research questions regarding families with children in need of care, such as relationships between individual needs and existing range of services. Therefore, a structured assessment (family-reported needs) is to be developed as well as a handbook (transfer model) with recommendations for the implementation of sustainable processes and structures for the needs of families with children in need of care. Eventually, these results will be applied in the standard care to strengthen and relieve families with disabled or chronically ill children.

There are, however, a number of limitations. A possible limitation of this study is that we only use self-reported outcomes for the impact analysis of FGPs as our primary research goal. Outcomes reported by someone else (e.g. clinical data) are not planned. However, for the most part we use psychometrically tested instruments developed for different contexts and languages (incl. German). Compared to other studies using the measurements, we expect a slightly higher proportion of missing values in some items after 18 months, but tackling this issue with imputation techniques is feasible. Missing values in psychometric testing are not a problem per se, but may result in biased reliability scores [37]. Finally, the number of cases in this project is limited overall because the number of affected families and/or qualified professionals is comparatively small; not every affected family needs an FGP per se, and the offer is not sufficiently known.

## Supporting information

S1 Checklist SPIRIT 2013 checklist: Recommended items to address in a clinical trial protocol and related documents*. (DOC)

S1 File (PDF)

S1 Data (DOCX)

S2 Data (DOCX)
