# Supplementary material for: Family health partners in regional network structures (NEST): A non-randomized controlled trial among parents of chronically ill and disabled children
Source: PLoS One. 2023 Jul 17;18(7):e0288435. doi: 10.1371/journal.pone.0288435 (PMC10351712; doi:10.1371/journal.pone.0288435)
Supplement: S1 File — (PDF) [file pone.0288435.s002.pdf]

Kurzantragsformular

Lokale Ethikkommission der Psychologen am Universitätsklinikum Hamburg-Eppendorf

Studientitel	Studientitel (max. 256 Zeichen)			
Finanzierung des Projektes	Finanzierung			
Geplante Projektlaufzeit	von	tt.mm.jjjj	bis	tt.mm.jjjj
Onlinestudie	○ Ja		○ Nein	

Studienleiter/in

Prof. Dr. Olaf Von Dem Knesebeck
Martinistraße 52
20246 Hamburg
Institut für Medizinische Soziologie

Kooperationspartner/in

Name, Vorname	Nachname	Vorname
Straße, Hausnummer	Straßenname	Nr
Postleitzahl, Ort	12345	Wohnort

Kurze Beschreibung des Projektes (max. 500 Wörter) mit folgenden Gliederungspunkten:

- → Hintergrund
- → Vorgehen (Art und Umfang der Stichprobe, Rekrutierungswege, geplante Auswertung)
- → Erwarteter Nutzen, erwartete Ergebnisse

Hintergrund:

Vorgehen:

Erwarteter Nutzen:

Ethikantrag Ja Nein

1.a)	Wurde dieser Ethikantrag bereits durch eine andere Ethikkommission begutachtet?	○	○
1.b)	Liegt zu einer vergleichbaren Studie bereits ein positives Ethikvotum vor?	○	○

	Teilnehmer/inneninformation<br>Es erfolgt eine umfassende Aufklärung über	Ja	Nein
2.	... die Untersuchungsziele.	○	○
3.	... die wissenschaftliche Bedeutung der Studie, die den Aufwand rechtfertigt.	○	○
4.	... die Dauer der Untersuchung.	○	○
5.	... Belastungen und Risiken durch die Untersuchungsverfahren<br>(hier sollten psychische und physische Belastungen Erwähnung finden).	○	○
6.	... die Vergütung und andere Zusagen an die Teilnehmer/innen<br>(z.B. geplante Information über die finalen Forschungsergebnisse).	○	○
7.	... die Freiwilligkeit der Teilnahme.	○	○
8.	... die jederzeitige und folgenlose Möglichkeit die Teilnahme abzubrechen.	○	○

Diese Webseite verwendet Cookies

Wir verwenden Cookies, um Ihnen die Nutzung Ihres persönlichen Bereichs möglich zu machen. Sollten Sie mit der Nutzung von Cookies nicht einverstanden sein, kann unter Umständen die Nutzung des persönlichen Bereichs eingeschränkt sein. Bitte akzeptieren Sie die Verwendung von Cookies.
Weiterhin wird mit einem Klick auf "Speichern" ein Cookie erstellt, in dem Ihre Entscheidung gespeichert ist.

Wir möchten Sie ebenfalls darum bitten die Datenschutzerklärung dieser Webseite zur Kenntnis zu nehmen und zu akzeptieren.
Eine Benutzung aller interaktiven Elemente der Webseite ist ohne Zustimmung zur Datenschutzerklärung nicht möglich.
Ihre Zustimmung können Sie jederzeit über einen Link am unteren Ende der Datenschutzerklärung widerrufen.

Ja, ich akzeptiere Cookies auf dieser Webseite. ☑

Ja, ich habe die Datenschutzerklärung gelesen und akzeptiere sie. ☑

Speichern

Untersuchungsziele und –verfahren, manipulierte Rückmeldung über eigene Leistung etc.), bzw. im Falle einer absichtlichen Täuschung wird nach Beendigung der Studie umfassend über die wahren Untersuchungsziele aufgeklärt.

12. Die Teilnehmer/innen-Information ist allgemeinverständlich (Fachvokabular und Fremdwörter sind auf ein Minimum reduziert).

13. Sofern eine Rückmeldung von Befunden (z.B. aus dem MRT) an die Teilnehmer/innen vorgesehen ist, so wird dafür vor Beginn der Datenerhebung die Zustimmung der Teilnehmer/innen eingeholt.

14. Teilnehmer/innen (und ggf. zusätzlich deren gesetzliche Vertreter/in) erhalten eine Kopie (bei Online-Studien: Möglichkeit zum Ausdruck) der Aufklärung und Einverständniserklärung mit den Kontaktdaten des Studienleiters/ der Studienleiterin.

Freiwilligkeit der Teilnehmer/innen — Ja / Nein

15. Die Freiwilligkeit der Teilnahme ist gesichert.

16. Es werden nur einwilligungsfähige Personen untersucht, ansonsten wird das Einverständnis der gesetzlichen Vertreter/innen (z.B. Eltern, gesetzlicher Betreuer/gesetzliche Betreuerin) eingeholt.

17. Es besteht kein direktes Abhängigkeitsverhältnis zwischen Versuchsleiter/in/Studienleiter/in und Teilnehmer/in (z.B. Arzt/Ärztin-Patient/in, Dozent/in-Student/in).

18. Alle Personen sind über ihre Teilnahme informiert, d.h. es ist [illegible]

Diese Webseite verwendet Cookies

Wir verwenden Cookies, um Ihnen die Nutzung Ihres persönlichen Bereichs möglich zu machen. Sollten Sie mit der Nutzung von Cookies nicht einverstanden sein, kann unter Umständen die Nutzung des persönlichen Bereichs eingeschränkt sein. Bitte akzeptieren Sie die Verwendung von Cookies.
Weiterhin wird mit einem Klick auf "Speichern" ein Cookie erstellt, in dem Ihre Entscheidung gespeichert ist.

Wir möchten Sie ebenfalls darum bitten die Datenschutzerklärung dieser Webseite zur Kenntnis zu nehmen und zu akzeptieren. Eine Benutzung aller interaktiven Elemente der Webseite ist ohne Zustimmung zur Datenschutzerklärung nicht möglich.
Ihre Zustimmung können Sie jederzeit über einen Link am unteren Ende der Datenschutzerklärung widerrufen.

Ja, ich akzeptiere Cookies auf dieser Webseite. ☑

Ja, ich habe die Datenschutzerklärung gelesen und akzeptiere sie. ☑

Speichern

das im Alltag übliche Maß hinaus körperlich beansprucht (z.B. Medikamentengabe, invasive Messungen, Ausbelastungstest, ungewohnte Umweltbedingungen wie Hypoxie).

20. Durch die Studie werden die Teilnehmer/innen nicht psychisch über das im Alltag übliche Maß hinaus beansprucht (z.B. durch Tätigkeitsdauer, stark aversive Reize, Fragen nach illegalem Verhalten).

21. Im Falle einer besonderen Beanspruchung (Punkte 19, 20) werden die Teilnehmer/innen während und nach der Studie bei Bedarf intensiv betreut.

22. Die Teilnehmer/innen geben keine vertraulichen Informationen preis oder wurden – falls solche Informationen erfasst werden – vor Unterzeichnung der Einverständniserklärung darüber informiert.

Datenschutz	Ja	Nein

23. Es sind keine Video- oder Tonaufnahmen oder andere Verhaltensregistrierungen vorgesehen, welche eine eindeutige Identifizierung der Teilnehmer/innen durch Dritte möglich machen könnten.

24. Die Daten werden vollständig anonymisiert (so dass keine Zuordnung der Daten zu Personen möglich ist) oder pseudonymisiert (Speicherung der Daten mit einem Personencode, Daten und Namen werden in getrennten Dateien gespeichert).

25. Es ist sichergestellt, dass nur schweigeverpflichtete Personen Zugriff zu den persönlichen Daten haben (z.B. Aufbewahrung in einem verschlossenen Schrank, passwortgeschützte

Diese Webseite verwendet Cookies

Wir verwenden Cookies, um Ihnen die Nutzung Ihres persönlichen Bereichs möglich zu machen. Sollten Sie mit der Nutzung von Cookies nicht einverstanden sein, kann unter Umständen die Nutzung des persönlichen Bereichs eingeschränkt sein. Bitte akzeptieren Sie die Verwendung von Cookies.
Weiterhin wird mit einem Klick auf "Speichern" ein Cookie erstellt, in dem Ihre Entscheidung gespeichert ist.

Wir möchten Sie ebenfalls darum bitten die Datenschutzerklärung dieser Webseite zur Kenntnis zu nehmen und zu akzeptieren. Eine Benutzung aller interaktiven Elemente der Webseite ist ohne Zustimmung zur Datenschutzerklärung nicht möglich.
Ihre Zustimmung können Sie jederzeit über einen Link am unteren Ende der Datenschutzerklärung widerrufen.

Ja, ich akzeptiere Cookies auf dieser Webseite. ☑

Ja, ich habe die Datenschutzerklärung gelesen und akzeptiere sie. ☑

Speichern

(DGSVO-https://dsgvo-gesetz.de/) sind bekannt und werden eingehalten.

Fallzahlplanung		Ja	Nein
29.	Es wurde eine Fallzahlplanung durchgeführt.	○	○

Arzneimittelgesetz oder Medizinproduktegesetz		Ja	Nein
30.	Die vorgeschlagene Studie fällt nicht unter das Arzneimittel- oder das Medizinproduktegesetz.	○	○

Proband/innenhonorar		Ja	Nein
31.	Den Teilnehmenden wird eine finanzielle Vergütung gezahlt, die einen Durchschnittsbetrag von 10 Euro/Stunde nicht deutlich überschreitet.	○	○

Wegeversicherung		Ja	Nein
32.	Für die Teilnehmenden besteht eine Wegeversicherung oder die Teilnehmer/innen werden darüber aufgeklärt, dass der Anfahrtsweg nicht versichert ist.	○	○

Onlinestudien		Ja	Nein
33.	Die Teilnehmer/innen erhalten vor Beginn der eigentlichen Studie eine umfassende Teilnehmer/innen-Information und [illegible]	○	○

Diese Webseite verwendet Cookies

Wir verwenden Cookies, um Ihnen die Nutzung Ihres persönlichen Bereichs möglich zu machen. Sollten Sie mit der Nutzung von Cookies nicht einverstanden sein, kann unter Umständen die Nutzung des persönlichen Bereichs eingeschränkt sein. Bitte akzeptieren Sie die Verwendung von Cookies.
Weiterhin wird mit einem Klick auf "Speichern" ein Cookie erstellt, in dem Ihre Entscheidung gespeichert ist.

Wir möchten Sie ebenfalls darum bitten die Datenschutzerklärung dieser Webseite zur Kenntnis zu nehmen und zu akzeptieren.
Eine Benutzung aller interaktiven Elemente der Webseite ist ohne Zustimmung zur Datenschutzerklärung nicht möglich.
Ihre Zustimmung können Sie jederzeit über einen Link am unteren Ende der Datenschutzerklärung widerrufen.

Ja, ich akzeptiere Cookies auf dieser Webseite. ☑

Ja, ich habe die Datenschutzerklärung gelesen und akzeptiere sie. ☑

Speichern

36. Die Teilnehmer/innen-Information enthält Ansprechpartner/innen für die Teilnehmer/innen (mit Kontaktdetails wie Telefonnummer oder Email-Adresse), und diese sind zeitnah verfügbar.

37. Der Teilnehmer/die Teilnehmerin wird aufgefordert, die Teilnehmer/innen-Information auszudrucken.

38. Die Einhaltung von Einschluss- und Ausschlusskriterien ist sichergestellt (z.B. über Filter).

39. Es werden keine IP-Adressen der Teilnehmer/innen gespeichert.

40. Email-Adressen werden nicht erfragt, oder es werden kostenlose Möglichkeiten benannt, eine anonymisierte Email-Adresse zu generieren.

41. Sollte die Erstellung eines Codewortes notwendig sein, wird den Teilnehmer/innen eine Vorgehensweise ähnlich der DGPs-Empfehlung vorgeschlagen (www.dgps.de).

42. Der Provider der Online-Plattform erkennt europäische Datenschutzrichtlinien an, oder der Server steht in Deutschland und unterliegt den in Deutschland üblichen Sicherheitsstandards.

Genauere Informationen zu einzelnen Themen und zum Format der Verfassung eines Vollantrages können der folgenden Internetseite der Ethikrichtlinien der Deutschen Gesellschaft für Psychologie (DGPs) entnommen werden: www.dgps.de/index.php?id=185.

Datenschutzrechtliche Aspekte von Forschungsvorhaben werden durch die Ethikkommission grundsätzlich nur kursorisch geprüft. Diese Bewertung ersetzt mithin nicht die Konsultation des/der zuständigen betrieblichen oder behördlichen Datenschutzbeauftragten.

Diese Webseite verwendet Cookies

Wir verwenden Cookies, um Ihnen die Nutzung Ihres persönlichen Bereichs möglich zu machen. Sollten Sie mit der Nutzung von Cookies nicht einverstanden sein, kann unter Umständen die Nutzung des persönlichen Bereichs eingeschränkt sein. Bitte akzeptieren Sie die Verwendung von Cookies.
Weiterhin wird mit einem Klick auf "Speichern" ein Cookie erstellt, in dem Ihre Entscheidung gespeichert ist.

Wir möchten Sie ebenfalls darum bitten die Datenschutzerklärung dieser Webseite zur Kenntnis zu nehmen und zu akzeptieren.
Eine Benutzung aller interaktiven Elemente der Webseite ist ohne Zustimmung zur Datenschutzerklärung nicht möglich.
Ihre Zustimmung können Sie jederzeit über einen Link am unteren Ende der Datenschutzerklärung widerrufen.

Ja, ich akzeptiere Cookies auf dieser Webseite. ☑

Ja, ich habe die Datenschutzerklärung gelesen und akzeptiere sie. ☑

Speichern

Universitätsklinikum
Hamburg-Eppendorf
(UKE)

Martinistraße 52
20246 Hamburg

E-Mail: LPEK@uke.de
Telefon: +49 (0) 40
7410 - 0

Hädel Computer Hardware

Diese Webseite verwendet Cookies

Wir verwenden Cookies, um Ihnen die Nutzung Ihres persönlichen Bereichs möglich zu machen. Sollten Sie mit der Nutzung von Cookies nicht einverstanden sein, kann unter Umständen die Nutzung des persönlichen Bereichs eingeschränkt sein. Bitte akzeptieren Sie die Verwendung von Cookies.
Weiterhin wird mit einem Klick auf "Speichern" ein Cookie erstellt, in dem Ihre Entscheidung gespeichert ist.

Wir möchten Sie ebenfalls darum bitten die Datenschutzerklärung dieser Webseite zur Kenntnis zu nehmen und zu akzeptieren. Eine Benutzung aller interaktiven Elemente der Webseite ist ohne Zustimmung zur Datenschutzerklärung nicht möglich.
Ihre Zustimmung können Sie jederzeit über einen Link am unteren Ende der Datenschutzerklärung widerrufen.

Ja, ich akzeptiere Cookies auf dieser Webseite. ☑

Ja, ich habe die Datenschutzerklärung gelesen und akzeptiere sie. ☑

Speichern
