# Supplementary material for: Family health partners in regional network structures (NEST): A non-randomized controlled trial among parents of chronically ill and disabled children
Source: PLoS One. 2023 Jul 17;18(7):e0288435. doi: 10.1371/journal.pone.0288435 (PMC10351712; doi:10.1371/journal.pone.0288435)
Supplement: S1 Data — (DOCX) [file pone.0288435.s003.docx]

**Short Application Form**

**Framework data (title of the project, project management, financing, etc.)**

Title: Strengthening and relief for families with children in need of care through family health partners (FGP) in regional NEtwork STructures (NEST)

Project managemnt: aQua-Institute for Applied Quality Promotion and Research in Healthcare GmbH, Goettingen

Funding: Innovation Fund of the Federal Joint Committee (G-BA), Berlin (grant number: 01VSF20004)

**Brief description of the project (max. 500 words)**

Background

Families with children with severely impaired health are exposed to particular emotional, social, economic and temporal burdens. This results in further risks, which often leads to a poor living and care situation for the affected families. This is also attributed to legal regulations that do not fit to the respective individual life situation and the lack of competent counseling services.

Objectives

The overarching project goal is to evaluate the effectiveness of a professional support function that works across sectors and service providers for families with children in need of care. The network support provided by so-called FamilienGesundheitsPartner (FGP) aims to provide all members of families with children in need of care with individual care that is tailored to their needs. The medium-term goal of FGP support is to support the family as a self-help system, i.e. to strengthen or maintain them as a self-competent, independently acting and primary resource for the care and support of their children in need of care

Instruments

The analysis of the effectiveness of this intervention primarily uses psychometrically validated instruments on the following topics:

- mental and physical health (e.g. SF-12)

- well-being/quality of life (e.g. WHO-5)

- family burdens (e.g. FaBel-20)

- resilience (e.g. BRC)

- resilience factors (e.g. SOP2)

- social support (e.g. OSSS-3)

In addition, the follolwing data is collected:

- socio-demographic and socio-economic information

- need for care and support (age of the child, degree of care, etc.)

- use of relevant support and counceling services.

Procedure (type/size of sample, recruitment, planned analysis)

A longitudinal case-control study (quantitative online survey with SoSci Survey) is planned over 18 months with four measurement points (t0 at the beginning of the intervention, interim evaluation after 6 (t1) and 12 (t2) months and a final survey after 18 months (t3). The estimated approximately 100 families per IG and CG are recruited via network partners in three regions (Greater Trier area, Greater Saarbrücken area, Munich). The case number planning was based on an alpha level of .05 with a test power of .80 and a medium effect size (Cohen's d). The families are recruited for the CG via the Kindernetzwerk e.V., which also has regional associations in these regions, and through increased public relations work.

Mixed regression models (multilevel models or mixed effects models) should be used for the analysis of the longitudinal data. The data is cleaned and processed with R and SPSS from version 26. Post-coding/categorization of the free text answers and free text fields is carried out by student assistants. All variables with a personal identification risk are anonymised, coarsened or removed.

Expected benefits/results

Since the FGP is implemented in a support structure that has been established for many years, the findings will definitely lead to the improvement and further development of regional care. A manual with implementable recommendations for comparable supply and support networks will be created explicitly during the project period. The results of the study are significant both for the affected families themselves and for the political decision-making processes. In addition, publications and lectures in a scientific context will contribute to the dissemination of knowledge in this field.

**Checklist (with comments if "no")**

- 13: Statement not applicable. There are no links with other participants.
- 21: Statement not applicable, there is no particular stress.
- 26: The data is collected in pseudonymised form, so that the reference to the possibility of deleting the data does not appear to make sense.
- 31: A small expense allowance for the families from the CG is planned, since they do not (yet) benefit from the intervention.
- 32: There are no access routes.
- 41: A code word is required for mapping the longitudinal section data.
