# Supplementary material for: Family health partners in regional network structures (NEST): A non-randomized controlled trial among parents of chronically ill and disabled children
Source: PLoS One. 2023 Jul 17;18(7):e0288435. doi: 10.1371/journal.pone.0288435 (PMC10351712; doi:10.1371/journal.pone.0288435)
Supplement: S2 Data — (DOCX) [file pone.0288435.s004.docx]

**Kurzantragsformular**

**Rahmendaten (Titel des Projekts, Projektleitung, Finanzierung etc.)**

Titel: Stärkung und Entlastung von Familien mit pflegebedürftigen Kindern durch Familien-Gesundheits-Partner (FGP) in regionalen NEtzwerk-Strukturen (NEST)

Projektleitung: aQua-Institut für angewandte Qualitätssicherung und Forschung im Gesundheitswesen GmbH, Göttingen

Finanzierung: Innovationsfonds des Gemeinsamen Bundesausschusses (G-BA), Berlin
(Fkz.: 01VSF20004).

**Kurze Beschreibung des Projekts (max. 500 Wörter)**

Hintergrund

Familien mit gesundheitlich stark beeinträchtigten Kindern sind besonderen emotionalen, sozialen, ökonomischen und zeitlichen Belastungen ausgesetzt. Hieraus resultieren weitere Risiken, was häufig zu einer schlechten Lebens- und Versorgungssituation der betroffenen Familien führt. Dies wird u. a. auch auf gesetzliche Regelungen, die den jeweiligen individuellen Lebenssituationen nicht gerecht werden, und das Fehlen von kompetenten Beratungs- und Anlaufstellen zurückgeführt.

Ziele

Übergeordnetes Projektziel ist die Evaluation der Wirksamkeit einer sektoren- und leistungsträgerübergreifend agierenden professionellen Unterstützungsfunktion für Familien mit pflegebedürftigen Kindern. Die Netzwerk-Unterstützung durch sog. FamilienGesundheitsPartner (FGP) zielt auf eine bedarfs- und bedürfnisgerechte, individuelle Versorgung aller Mitglieder in Familien mit pflegebedürftigen Kindern ab. Mittelfristiges Ziel der FGP-Unterstützung ist es, die Familie als Selbsthilfesystem, d. h. als selbstkompetente, eigenständig agierende und primäre Ressource der Versorgung und Unterstützung ihrer pflegebedürftigen Kinder zu stärken oder zu erhalten.

Instrumente

Eingesetzt werden bei der Wirksamkeitsanalyse dieser Intervention v.a. psychometrisch validierte Instrumente zu folgenden Themen:
- psychische und physische Gesundheit (z.B. SF-12)
- Wohlbefinden/Lebensqualität (z.B. WHO-5)
- familiäre Belastungen (z.B. FaBel-15)
- Resilienz (z.B. BRC)
- Resilienzfaktoren (z.B. SOP2)
- soziale Unterstützung (z.B. OSSS-3)
Darüber hinaus werden
- soziodemografische und -ökonomische Angaben
- der Pflege- und Betreuungsbedarf (Alter des Kindes, Pflegegrad etc.) sowie
- Daten zur Inanspruchnahme relevanter Unterstützungs- und Beratungsangebote
erhoben.

Vorgehen (Art/Umfang der Stichprobe, Rekrutierung, geplante Auswertung)

Geplant ist eine longitudinale Fall-Kontroll-Studie (quantitative Online-Befragung mit SoSci Survey) über 18 Monate mit vier Messzeitpunkten (T0 zu Beginn der Intervention, Zwischenevaluation nach 6 (T1) und 12 (T2) Monaten sowie eine Abschlussbefragung nach 18 Monaten (T3). Die Rekrutierung der geschätzten ca. 100 Familien je IG und KG erfolgt über Netzwerk-Partner in drei Regionen (Großraum Trier, Großraum Saarbrücken, München). Grundlage der Fallzahlplanung waren ein Alpha-Niveau von 0,05 bei einer Teststärke von 0,80 sowie eine mittlere Effektstärke (Cohens d). Über das Kindernetzwerk e.V., das in diesen Regionen ebenfalls über regionale Verbände verfügt, sowie über verstärkte Öffentlichkeitsarbeit werden die Familien für die KG rekrutiert.

Für die Analyse der Längsschnittdaten sollen gemischte Regressionsmodelle (Mehrebenenmodelle oder mixed effects models) verwendet werden. Die Datenbereinigung und -aufbereitung erfolgen mit R und SPSS ab Version 26. Eine Nachkodierung/Kategorisierung der Freitextantworten und Freitextfelder erfolgt durch studentische Hilfskräfte. Alle Variablen mit Personenidentifikationsrisiko werden anonymisiert, vergröbert oder entfernt.

Erwarteter Nutzen/erwartete Ergebnisse

Da der FGP in einer langjährig etablierten Unterstützungsstruktur implementiert wird, werden die Erkenntnisse in jedem Fall zur Verbesserung und Weiterentwicklung der regionalen Versorgung führen. Ein Handbuch mit umsetzbaren Empfehlungen für vergleichbare Versorgungs- und Unterstützungsnetzwerke wird explizit im Rahmen der Projektlaufzeit erstellt. Die Ergebnisse der Studie sind bedeutsam sowohl für die betroffenen Familien selbst als auch für die politischen Entscheidungsprozesse. Darüber hinaus werden Publikationen und Vorträge im wissenschaftlichen Kontext zur Verbreitung des Erkenntnisstands in diesem Feld beitragen.

**Checkliste (mit Kommentaren bei „nein“)**

- 13: Aussage nicht zutreffend. Es gibt keine Verknüpfungen mit anderen Beteiligten.
- 21: Aussage nicht zutreffend, es liegt keine besondere Beanspruchung vor.
- 26: Die Daten werden pseudonymisiert erhoben, so dass der Hinweis auf die Möglichkeit der Löschung der Daten nicht sinnvoll erscheint.
- 31: Eine geringe Aufwandsentschädigung für die Familien aus der KG ist geplant, da diese (noch) nicht von der Intervention profitieren.
- 32: Es gibt keine Anfahrtswege.
- 41: Ein Codewort ist für die Zuordnung der Längsschnittdaten erforderlich.
